# Protective Effects of Cinnamaldehyde Against Heat Stress in Mice and Its Regulatory Influence on Estrogen Secretion

**DOI:** 10.3390/ani16213512

**Published:** 2026-11-06

**Authors:** Yuxin Guo, Yang Yang, Yunyi Zhang, Dengfeng Wang, Haiyan Wu, Panpan Guo, Yutong Yan, Guangwen Yin, Qingrui Meng, Lei Wang

**Affiliations:** 1 College of Animal Science, Fujian Agriculture and Forestry University, Fuzhou 350002, China; 2 College of Agriculture and Food Science, Jinshan College of Fujian Agriculture and Forestry University, Fuzhou 350002, China

**Keywords:** heat stress, cinnamaldehyde, mouse follicular granulosa cells, estrogen, steroidogenic-related genes

## Abstract

Heat stress (HS) causes reproductive damage in female mammals, including pigs, cattle, and sheep. As the primary site of estrogen (E_2_) biosynthesis, follicular granulosa cells (GCs) act as the critical target of HS injury. Previous studies have shown that cinnamaldehyde (CA) effectively participates in reproductive regulation in mice. Therefore, female mice and their follicular GCs were taken as the research subjects in this study. First, we adopted ELISA and H&E staining to investigate the effects of CA on ovarian follicular histology and E_2_ secretion in mice. Second, CCK-8 assay, flow cytometry, and ELISA were applied to clarify how CA regulates proliferation, apoptosis, and E_2_ secretion of mouse follicular GCs under HS. Finally, RT-qPCR and Western blotting (WB) were used to elucidate the protective effects of CA together with its underlying molecular mechanisms. Animal experiment results revealed that HS elevated the proportion of primordial and primary follicles and suppressed E_2_ secretion. Oral gavage of mice with CA at 25 mg/kg exhibited no obvious hepatotoxicity. This dosage effectively increased the percentage of secondary and antral follicles, alleviated HS-induced histological damage in ovaries, and raised serum E_2_ levels both under normal conditions and HS. Further in vitro cell experiments confirmed that HS reduced cell viability and accelerated apoptosis in mouse follicular GCs. HS decreased estrogen secretion by downregulating the relative protein expression of StAR and CYP19A1. Treatment with CA at a concentration of 2.5 × 10^−5^ M significantly increased cell viability and inhibited excessive apoptosis in mouse follicular GCs under HS. Under HS conditions, CA treatment elevated E_2_ secretion in mouse follicular GCs by upregulating the relative protein expression of StAR and CYP19A1. In conclusion, the findings of this study indicate that CA holds promise as a natural anti-HS additive.

## Simple Summary

Heat stress (HS) can induce reproductive damage in female mammals. Follicular granulosa cells (GCs) serve as the critical target of HS injury. Previous studies have shown that CA can effectively participate in reproductive regulation in mice. Therefore, to clarify the alleviating effect and regulatory mechanism of CA against HS in female mice, this study treated HS female mice and their follicular GCs with cinnamaldehyde. Animal experiments showed that intragastric administration of CA at 25 mg/kg caused no obvious hepatotoxicity. This dosage effectively ameliorated HS-induced ovarian histological injury and elevated serum E_2_ concentrations under both normal and HS conditions. Further in vitro cellular assays verified that treatment with 2.5 × 10^−5^ M CA markedly increased cell viability and suppressed excessive apoptosis in mouse follicular GCs exposed to HS. Under HS, CA boosted E_2_ secretion in mouse follicular GCs by upregulating the protein expression of StAR and CYP19A1. In conclusion, CA exhibits great potential in alleviating HS in female mice.

## 1. Introduction

With the frequent occurrence of extreme high-temperature weather worldwide, heat stress (HS) has become a critical environmental factor restricting the sustainable development of animal husbandry in summer [1,2]. HS severely impairs the production performance and reproductive efficiency of economically important livestock, including dairy cows, pigs, and sheep, greatly hindering the development of animal husbandry [3,4,5]. In female mammals, HS damages the reproductive system, leading to estrous cycle disorders, arrested follicular development, abnormal early embryonic development, and other adverse outcomes [6,7]. Granulosa cells (GCs) of ovarian follicles constitute an important somatic cell population in the female mammalian reproductive system and serve as the primary site for estrogen (E_2_) synthesis [8]. Accumulating evidence has demonstrated that HS triggers oxidative stress in GCs, markedly elevates the apoptosis rate of GCs, and consequently induces follicular atresia [6,9]. At present, maintaining an appropriate ambient temperature in livestock houses and ensuring an adequate water supply remain vital approaches to avoid HS in animals. Nevertheless, these strategies are constrained by site costs and extreme weather conditions [10]. Therefore, exploring natural active substances capable of effectively alleviating HS injury is regarded as an effective regulatory strategy [11,12]. This is of great significance for mitigating the adverse impacts of high temperatures in summer on the reproductive performance of female animals.

Cinnamaldehyde (CA) is the principal active ingredient extracted from cinnamon bark and classified as an aromatic aldehyde compound [13]. Its molecular structure consists of a benzene ring, an acrolein side chain, and an aldehyde group [13]. The α, β-unsaturated carbonyl group in its structure serves as the key functional group responsible for its biological activities [13]. Maintaining the homeostasis of reproductive hormones is an important entry point for alleviating HS [14,15]. StAR and CYP11A1 are key enzymes in the biosynthesis of steroid reproductive hormones. Our lab’s earlier research verified that CA can effectively upregulate the transcriptional and protein expression levels of CYP11A1 and StAR in male mice, which lays a theoretical foundation for further exploring the protective effects of CA against HS in female mammals [16].

In this study, we investigated the protective effects of CA on heat-stressed mice and their follicular GCs. We elucidated the protective effects and molecular mechanisms of CA on the E_2_ synthesis function of mouse follicular GCs. This study provides a theoretical basis for the development of CA as a natural anti-HS additive.

## 2. Materials and Methods

### 2.1. Animals

Three-week-old female SPF KM mice were purchased from SiPeiFu Biotechnology Co., Ltd. (Suzhou, China). The animal experiment was approved by the Ethics Committee of Fujian Agriculture and Forestry University (Fuzhou, China, Approval No.: PZCASFAFU24041) on 15 March 2023.

### 2.2. Experimental Grouping

For dosage screening for the in vivo animal study, 25 female 3-week-old KM mice of comparable body weight were randomly divided into 5 groups (*n* = 5): CON group (1 × PBS), DMSO group, and intragastric CA treatment groups (12.5 mg/kg, 25 mg/kg, 37.5 mg/kg). Sixty female 3-week-old KM mice were randomly allocated to 4 groups for the animal therapeutic experiment (*n* = 15): CON group (1 × PBS, 25 °C), HS group, HS-DMSO group, and HS-CA group. All mice were housed under natural light conditions with free access to food and water. Animals were fasted for 2 h before intragastric gavage. Each group received the corresponding intragastric treatment. HS treatment involved placing the mice in a 40 °C environment for 2 h. Body weight (*n* = 5) and E_2_ levels of mice (*n* = 3) were measured at three time points: week 1, week 2, and week 3.

To screen the optimal CA treatment concentration for follicular GCs under HS, mouse follicular GCs were divided into the CON group and CA treatment groups (1.25 × 10^−5^ M, 2.5 × 10^−5^ M, 3.75 × 10^−5^ M, and 5 × 10^−5^ M). After the optimal concentration was determined, mouse follicular GCs were assigned to four groups for subsequent experiments: CON group, CA group, HS group, and HS+CA group. The final concentration of solvent DMSO was 0.1%. The cell experiment was performed with three biological replicates, and three technical replicates were conducted for each sample.

### 2.3. Isolation and Identification of Mouse Follicular Granulosa Cells

The isolation method of mouse follicular GCs was referenced from Li et al. [17]. Mice (3-week-old) received an intraperitoneal injection of 10 IU pregnant mare serum gonadotropin (PMSG) to induce superovulation. After 40–48 h, mice were anesthetized with ether and sacrificed via cervical dislocation. Ovaries were harvested under sterile conditions. After being rinsed twice with PBS, ovaries were transferred into pre-cooled handling medium. Follicles were punctured to release GCs and cumulus–oocyte complexes (COCs). Follicular fluid was collected, filtered through a 200-mesh cell strainer, and centrifuged; the supernatant was discarded. The cells were resuspended, seeded evenly into 6-well cell culture plates, and incubated at 37 °C with 5% CO_2_.

The isolated cells were seeded into 24-well cell culture plates with cell coverslips. Cells were fixed with 4% paraformaldehyde for 15 min and rinsed with PBS. After permeabilization with 0.3% Triton X-100 for 10 min, cells were washed with PBS. Subsequently, cells were blocked with 5% BSA at room temperature for 1 h, followed by PBS washing. Cells were incubated overnight at 4 °C with primary antibody (FSHR Rabbit pAb (A1480), ABclonal Biotechnology Co., Ltd., Wuhan, China, working concentration 1:200) against FSHR, followed by PBS washing. Fluorescently labeled secondary antibody (Goat Anti-Rabbit IgG H&L, ZenBio, Chengdu, China, working concentration 1:500) was added, and samples were incubated at room temperature in the dark for 1 h. After staining with DAPI at room temperature for 10 min, cells were rinsed with PBS. Anti-fade mounting medium was dropped onto the samples for coverslip sealing. Finally, cells were observed and photographed under an inverted fluorescence microscope.

### 2.4. Establishment of Heat Stress Model for Mouse Follicular Granulosa Cells

The procedure for establishing the cellular HS model was modified based on the protocol reported by Li et al. [17]. The isolated mouse follicular GCs were cultured in an incubator with 5% CO_2_ at 40 °C for 0 h, 6 h, 12 h, 24 h, and 48 h, respectively.

### 2.5. Estrogen Measurement

E_2_ levels are strongly associated with the estrous phase in mice. Therefore, we measured serum E_2_ levels only in mice in the estrus phase. Estrus staging was performed according to Zhou et al. [18]. Under the microscope, numerous anucleate keratinized squamous epithelial cells were observed, with very few leukocytes (shown in Figure S2). E_2_ levels in serum and culture supernatant of mouse follicular GCs were measured with a mouse estradiol ELISA kit (Shanghai Kexing Trading Co., Ltd., Shanghai, China) (n = 3). The detection range of the kit was 8–200 pmol/L. The coefficient of variation (CV) was less than 9% for intra-assay and less than 11% for inter-assay. Each sample was measured in triplicate wells, and the average value was taken.

### 2.6. Cell Counting Kit-8 (CCK-8) Assay

The isolated mouse follicular GCs were evenly seeded into a 96-well plate and cultured for 24 h. After cells were treated according to grouping, cell viability was determined using the CCK-8 assay. A refers to the OD value of treatment groups (heat-treated wells containing cells, culture medium, and CCK-8 reagent); B refers to the OD value of the control group (cells cultured at 37 °C with culture medium and CCK-8 reagent); C refers to the OD value of blank wells containing only culture medium and CCK-8 reagent.

$$Cell\;viability=\frac{A-C}{B-C}\times 100\%$$

### 2.7. Flow Cytometry

The isolated mouse follicular GCs were seeded into 6-well plates. When cell confluence reached approximately 70%, cells were subjected to heat treatment at 40 °C in a cell incubator for 24 h. Cell apoptosis was detected using the Annexin V-FITC/PI Apoptosis Detection Kit (Wuhan Elabscience Biotechnology Co., Ltd., Wuhan, China). Data acquisition and analysis were performed on a NovoCyte 1030 flow cytometer(ACEA BIO Co.,Ltd, Hangzhou, China) with its supporting software, as previously described [19].

### 2.8. Hematoxylin–Eosin (H&E) Staining

Mice were anesthetized with ether and euthanized by cervical dislocation. Liver and ovarian tissue samples were harvested from the mice and fixed in 4% paraformaldehyde. H&E staining was performed as previously described [16].

### 2.9. Follicle Classification and Counting

The histological characteristics of follicles at various stages were observed under a light microscope, and the classification criteria were based on the Lundy method [20], as shown in Table 1. Atretic follicles refer to follicles that arrest development and undergo degenerative apoptosis at any developmental stage and fail to reach ovulation. The identification of atretic follicles is primarily based on the morphology (apoptotic features) and laminar arrangement of granulosa cells [21]. The number of primordial/primary follicles was obtained from the single largest two-dimensional section, and the criterion that only follicles with a clearly observed oocyte nucleus were included in the count was strictly followed. For follicle counting, ovarian sections from 3 randomly selected mice in each group were used, and a total of 50 follicles were counted in the ovarian sections of each mouse. For statistical analysis, primordial and primary follicles were classified as stage A (early stage of follicular development), while secondary and antral follicles were grouped into stage B (late stage of follicular development).

### 2.10. RT-qPCR Analysis

The isolated mouse follicular GCs were seeded into 12-well plates. When cell confluence reached approximately 70%, cells were treated in accordance with grouping for 24 h. Total cellular RNA was extracted via the TRIzol method under RNase-free conditions. The concentration and purity of RNA were quantified with an Infinite M200 Pro multimode microplate reader(Tecan (Shanghai) Trading Co., Ltd., Shanghai, China), followed by reverse transcription. All primers were synthesized by Fuzhou Shangya Biotechnology Co., Ltd., Fuzhou, China), and the primer sequences are listed in Table 2. RT-qPCR detection was carried out with the NovoStart^®^ SYBR qPCR SuperMix Plus kit(Thermo Fisher Scientific Inc., Shanghai, China). The amplification procedure was set as follows: initial denaturation at 95 °C for 1 min, followed by 40 cycles of denaturation at 95 °C for 20 s and annealing-extension at 60 °C for 1 min. *β-actin* was selected as the reference gene. The experimental data were analyzed using the 2^−△△Ct^ method.

### 2.11. Western Blotting (WB)

Total protein from mouse ovarian tissue was extracted following the manufacturer’s instructions for the Total Protein Extraction Kit (Kaiji Biotechnology Co., Ltd., Yixing, China). After protein quantification, proteins were separated via 12% SDS-PAGE and then transferred onto PVDF membranes. WB was conducted according to Meng et al. [16]. Finally, chemiluminescence development and quantitative analysis were conducted. The CYP19A1 primary antibody was purchased from ABclonal Biotechnology Co., Ltd. (Wuhan, China) and used at a working concentration of 1:1000. The StAR primary antibody was purchased from Proteintech (Wuhan, China) and used at a working concentration of 1:500. HRP Goat Anti-Rabbit IgG (H&L) was purchased from Uelandy (Suzhou, China) and used at a working concentration of 1:10,000.

### 2.12. Statistical Analysis

Data analyses in this study were performed using Excel, GraphPad Prism 11.0.1, and SPSS 24 software. All in vitro experimental data are expressed as the mean ± SD of 3 independent experiments. The Shapiro–Wilk test was used to assess whether the data were normally distributed. The Brown–Forsythe test was used to assess the homogeneity of variance. One-way ANOVA combined with Tukey’s test was adopted for statistical comparison. Statistical significance was defined as * *p* < 0.05 and ** *p* < 0.01. Different lowercase letters indicated statistically significant differences between groups.

## 3. Results

### 3.1. Screening of Cinnamaldehyde Treatment Concentrations for Animal Experiments

**Figure 1:**
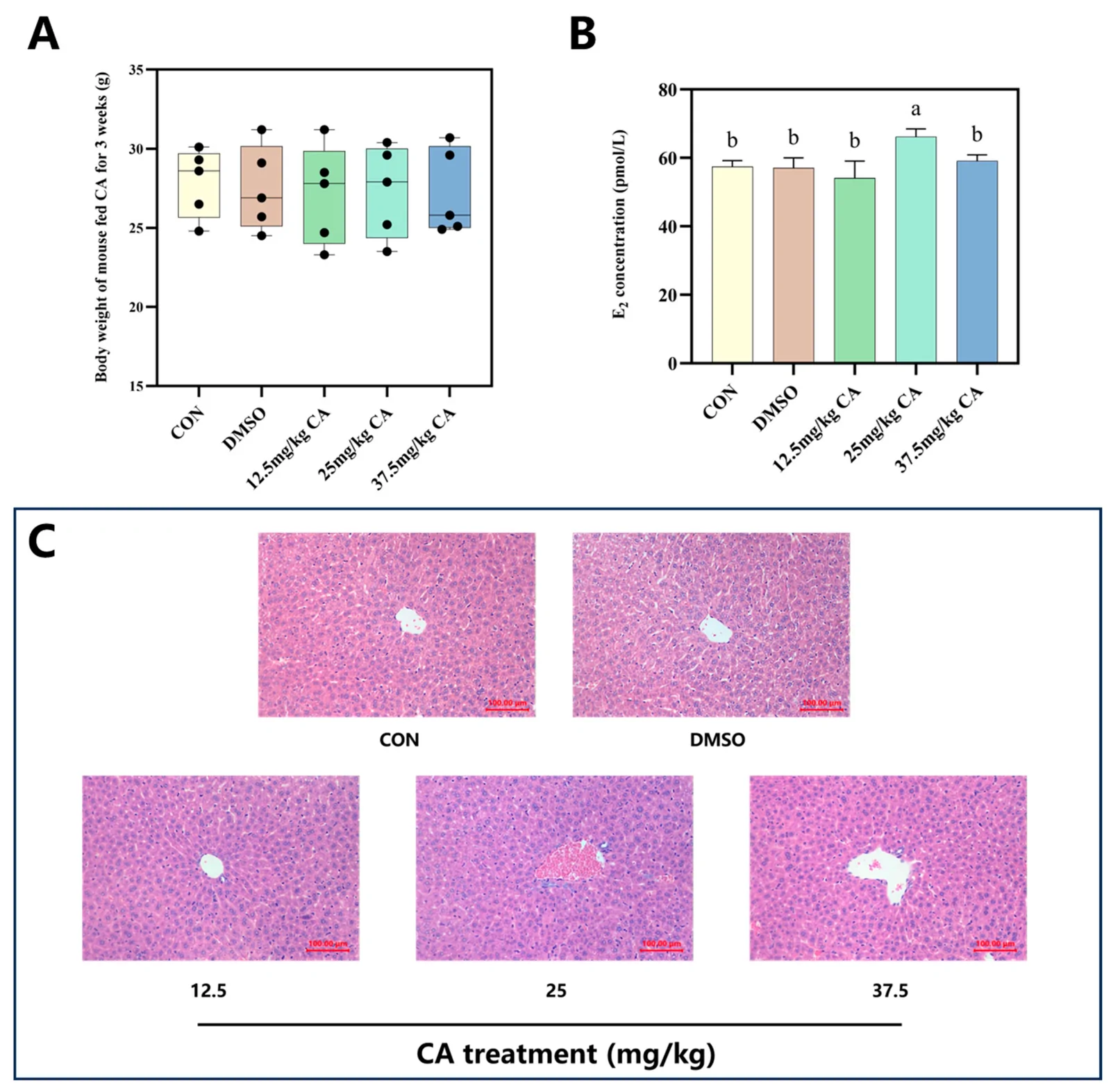
Results of CA treatment concentration screening: (**A**) Body weight of KM mice after 3 weeks of CA treatment (n = 5). (**B**) E_2_ secretion levels in KM mice following 3 weeks of CA treatment (n = 3). (**C**) H&E staining representative results of liver tissue sections from KM mice after 3 weeks of CA treatment (n = 3). Scale bar: 100 μm.

After three consecutive weeks of intragastric administration in mice, no significant differences in body weight were observed among all groups (Figure 1A). The serum E_2_ level in mice receiving the 25 mg/kg dosage was extremely significantly higher than that in the CON group (Figure 1B). The hepatic H&E staining results indicated that the liver tissue structure was intact in all groups, with hepatocytes arranged neatly in a radial pattern around the central vein, and no lipid droplet vacuolation or inflammatory injury was observed (Figure 1C). Hepatic H&E staining results revealed that no significant pathological alterations were observed within the tested dosage range of CA administration. Accordingly, the 25 mg/kg dosage was selected for subsequent experiments.

### 3.2. Effects of Cinnamaldehyde Treatment on Body Weight and Serum Estrogen Level in Heat-Stressed Mice

**Figure 2:**
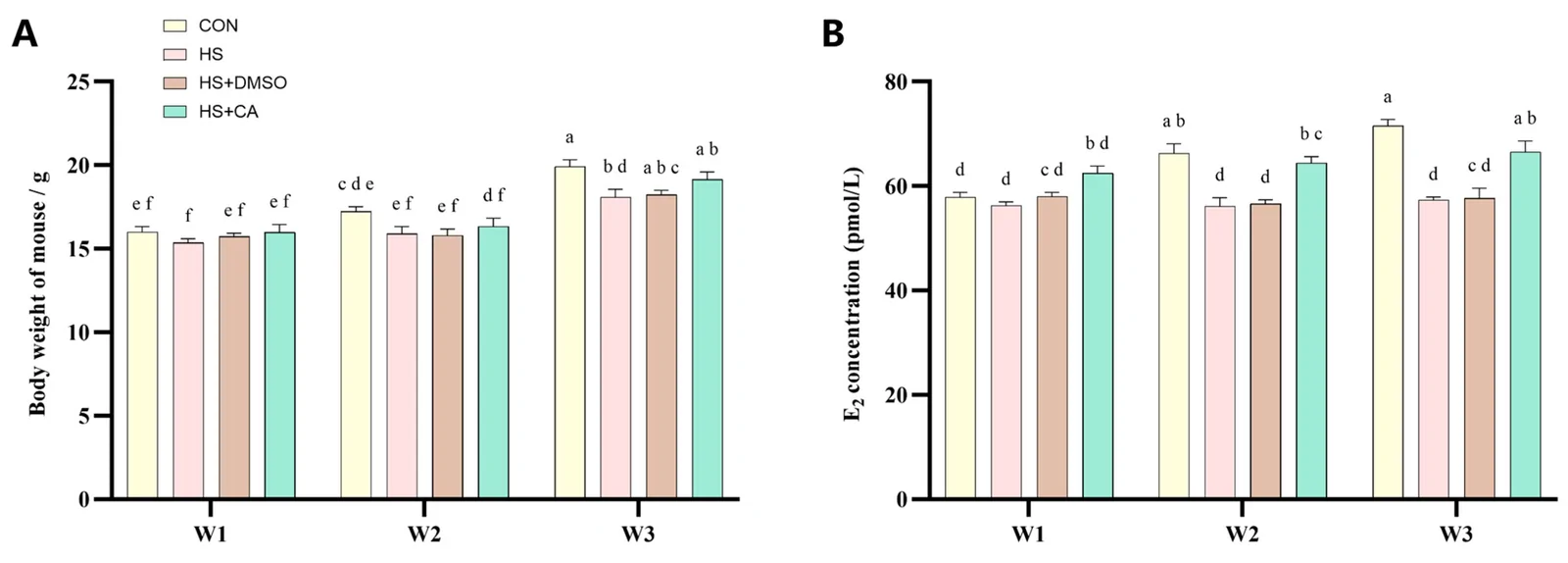
CA affects body weight and E_2_ levels in HS KM mice: (**A**) Effects of CA treatment for 1–3 weeks on body weight of HS KM mice (n = 5 at each time point). (**B**) Effects of CA treatment for 1–3 weeks on E_2_ levels in HS KM mice (n = 3 at each time point).

No significant differences in mouse body weight were found among all groups in Week 1 (W1) (Figure 2A). In Week 2 (W2), body weights of mice receiving chronic HS treatment showed a declining trend relative to the CON group, with no statistical significance (Figure 2A). In Week 3 (W3), body weights of the HS group and HS+DMSO group were significantly lower than those of the CON group (*p* < 0.05) (Figure 2A). The body weight of mice in the HS+CA group exhibited no statistically significant difference compared with all other groups; nevertheless, an upward trend was observed in the HS+CA group versus the HS group and HS+DMSO group (Figure 2A). The body weights of mice in all experimental groups increased over time (Figure 2A). During the W1–W3 period, compared with the HS group, the HS+CA group showed that CA intervention could effectively alleviate the inhibitory effect of HS on growth.

At W1, serum E_2_ concentrations in the 25 mg/kg CA treatment group were significantly higher than those in the CON group and HS group (*p* < 0.05) (Figure 2B). At W2 and W3, E_2_ levels in the HS group and HS+DMSO group decreased extremely significantly relative to the CON group (*p* < 0.01). CA treatment markedly elevated E_2_ concentrations under HS conditions (*p* < 0.05) (Figure 2B). Serum E_2_ levels in mice in the HS and HS+DMSO groups did not increase with the age of the mice. CA intervention could effectively alleviate this phenomenon.

### 3.3. Effects of Cinnamaldehyde Treatment on Ovarian Tissue Morphology of Heat-Stressed Mice

**Figure 3:**
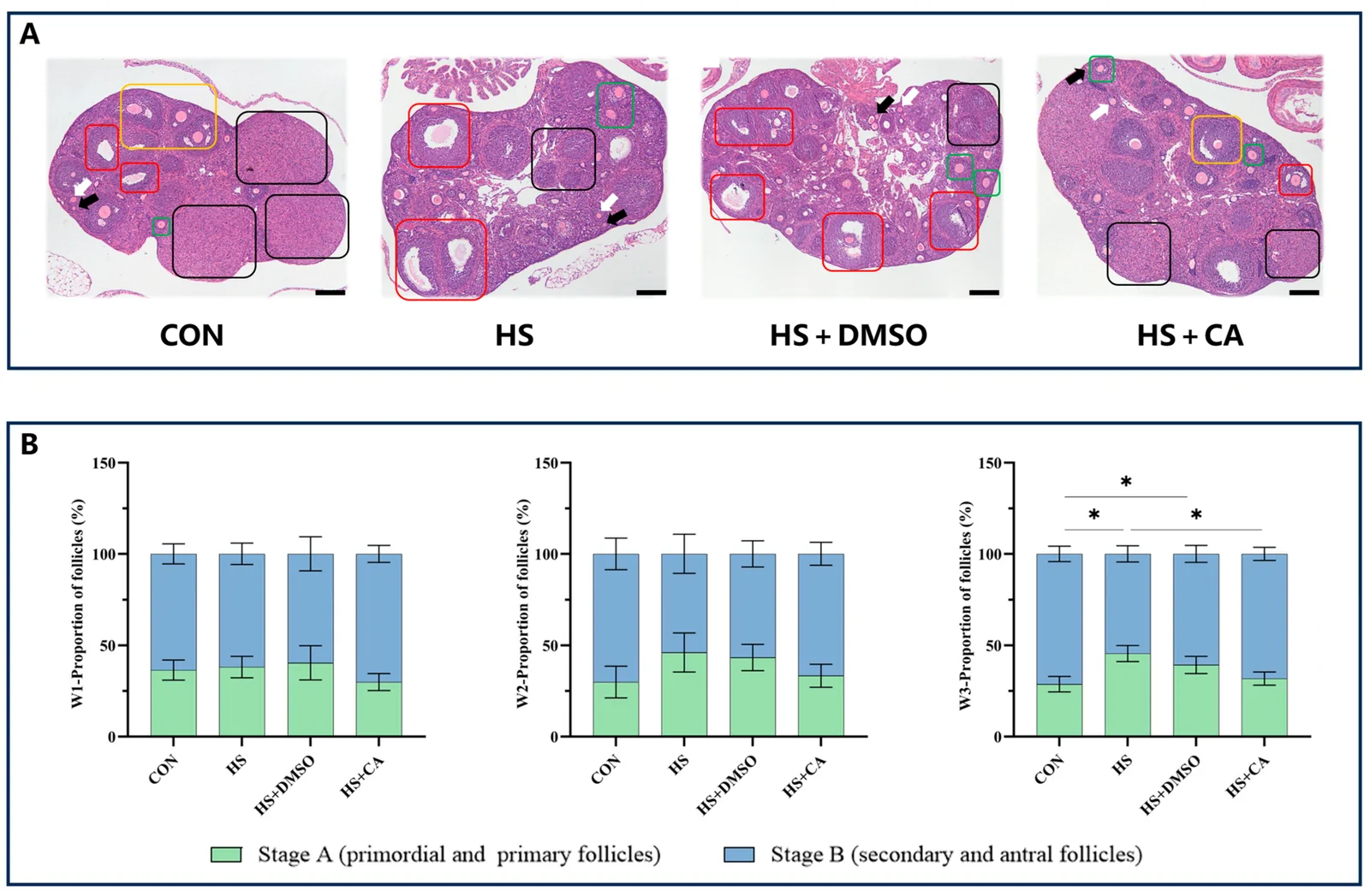
Effects of CA treatment on ovarian tissues of HS KM mice (n = 3): (**A**) Representative images of ovarian tissue sections; scale bar: 100 μm. (**B**) Statistical data for stage A and stage B follicles. Black arrows: primordial follicles. White arrows: primary follicles. Green square frames: secondary follicles. Yellow square frames: antral follicles. Black square frames: corpora lutea. Red square frames: atretic follicles.

Histological results are presented in Figure 3A. Primordial follicles are indicated by black arrows, and primary follicles by white arrows. The CON group and HS+CA group possessed abundant secondary (green square frames) and antral follicles (yellow square frames). The granulosa cell layers were thick, compact, and intensely stained. Large corpus lutea indicated successful ovulation in the ovaries (black square frames), indirectly demonstrating active overall ovarian function. Although multiple growing follicles existed in the HS group and HS+DMSO group, most follicles were atretic follicles (red square frames).

No significant differences were observed in the relative proportions of primordial plus primary follicles as well as secondary plus antral follicles in mouse ovaries among all groups at W1 and W2 (*p* > 0.05) (Figure 3B). With prolonged HS duration, the relative proportion of primordial and primary follicles was markedly higher in the HS and HS+DMSO groups than in the CON group at W3 (*p* < 0.05), while the percentage of secondary and antral follicles decreased significantly. The proportion of secondary and antral follicles rebounded significantly in the HS+CA group, showing obvious improvement compared with the HS group (*p* < 0.05) (Figure 3B).

### 3.4. Identification of Mouse Follicular Granulosa Cells

**Figure 4:**
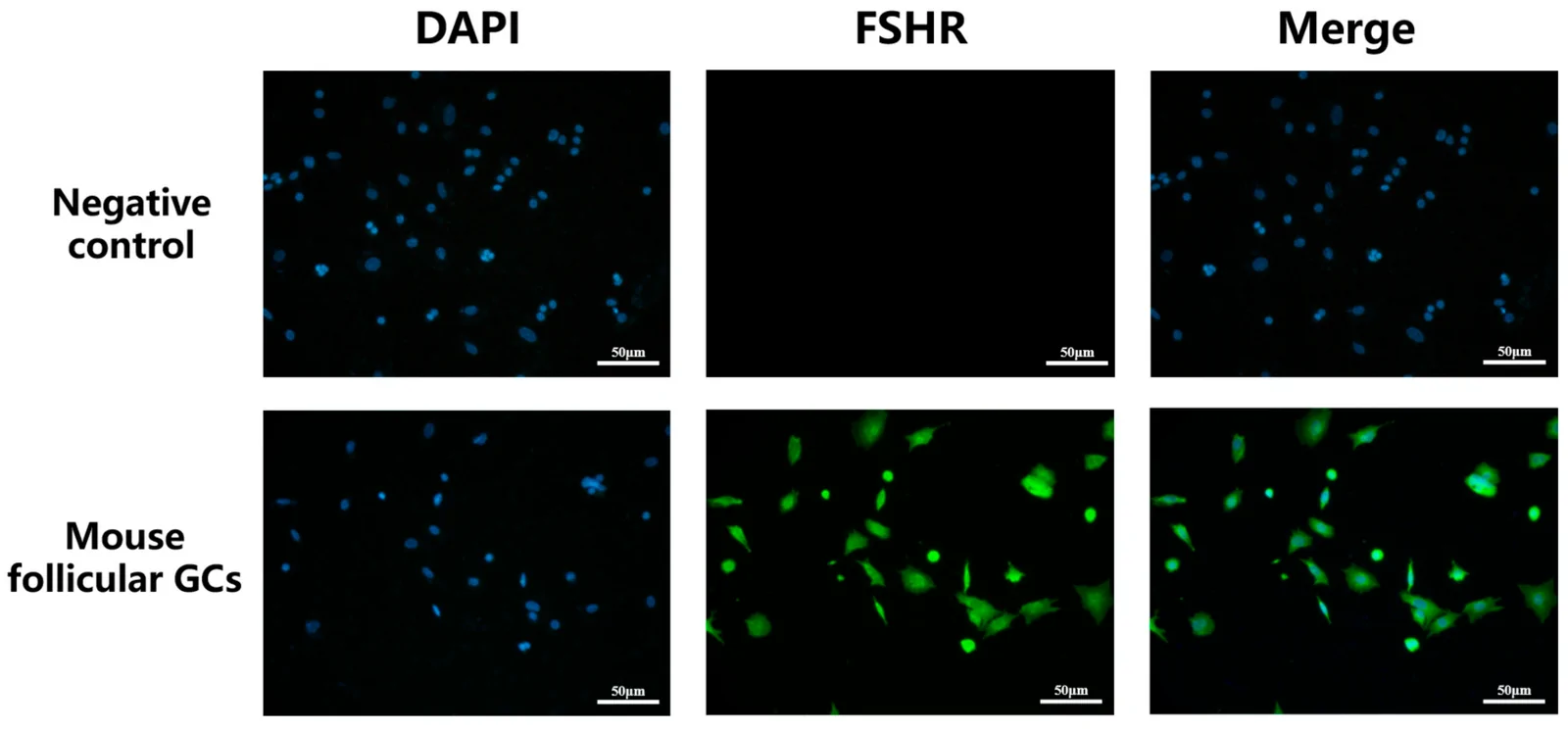
Immunofluorescence results of mouse follicular GCs (200×).

Mouse follicular GCs exhibited spindle-shaped adherent growth. The cell viability of passaged primary cells was relatively low. Therefore, freshly isolated mouse follicular GCs were directly used for all experiments after isolation and culture. Immunofluorescence staining was performed to detect the expression of FSHR on the cell membrane (Figure 4). The results verified that the isolated cells were mouse follicular GCs.

### 3.5. Establishment of Heat Stress Model of Mouse Follicular Granulosa Cells

**Figure 5:**
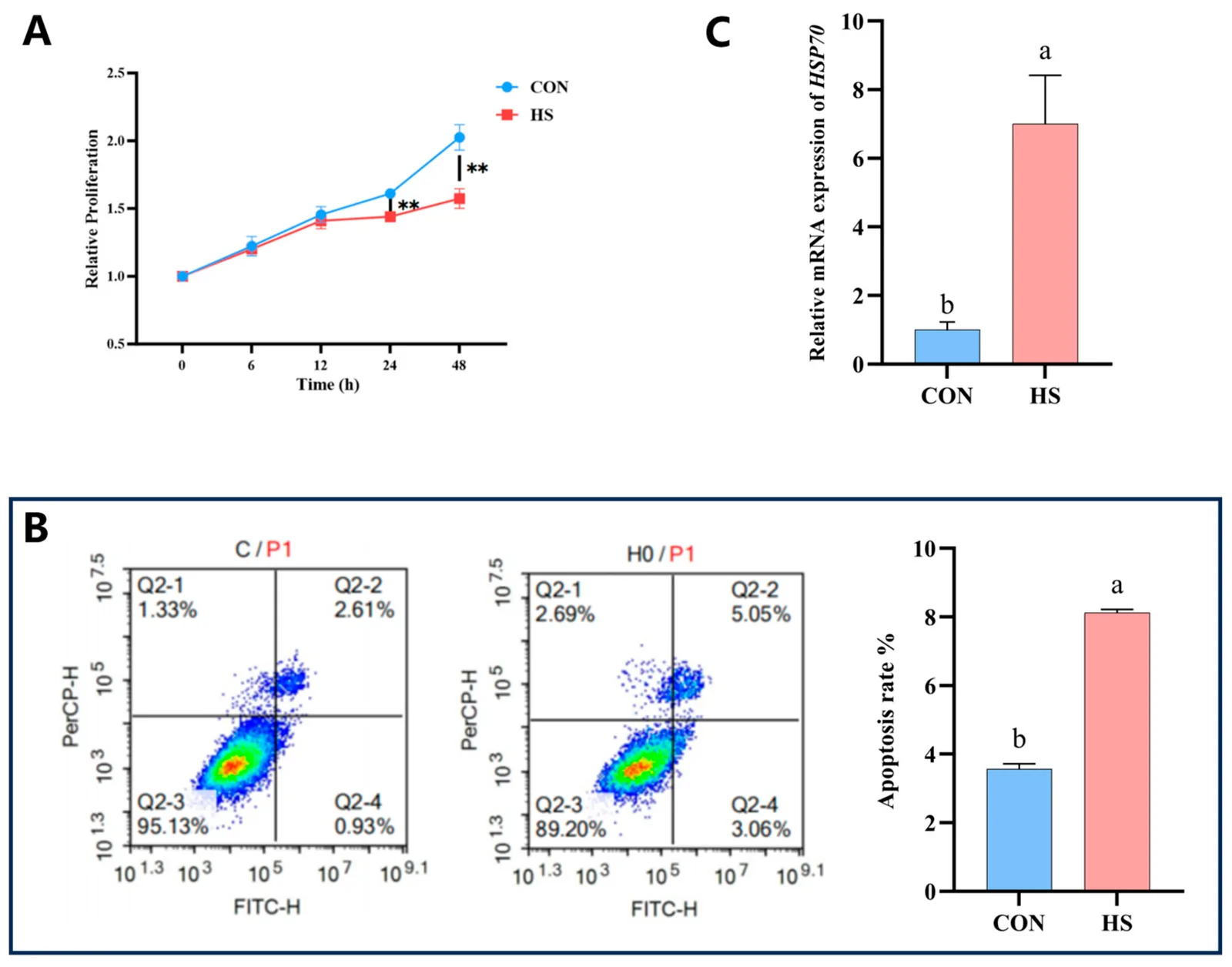
Establishment of the HS model in mouse follicular GCs: (**A**) Detection of cell viability via CCK-8 assay. (**B**) Determination of cell apoptosis by flow cytometry. Q2-1: necrotic cells or late apoptotic fragments; Q2-2: late apoptotic cells; Q2-3: live cells; Q2-4: early apoptotic cells. (**C**) Transcription level of the *HSP*70 gene.

After heat treatment at 40 °C for 24 h and 48 h, cell viability in the HS group decreased significantly compared with the CON group (Figure 5A). Accordingly, 40 °C heat treatment for 24 h was selected in this study to establish the cellular HS model.

Figure 5B reveals that the apoptosis rate of mouse follicular GCs was extremely significantly increased after heat treatment at 40 °C for 24 h (*p* < 0.01). Heat treatment at 40 °C for 24 h significantly upregulated the relative mRNA expression level of *HSP*70 (*p* < 0.05) (Figure 5C). Collectively, cellular HS models were successfully established in mouse follicular GCs via 24 h heat exposure at 40 °C.

### 3.6. Effects of Cinnamaldehyde Treatment on Viability and Apoptosis in Heat-Stressed Mouse Follicular Granulosa Cells

**Figure 6:**
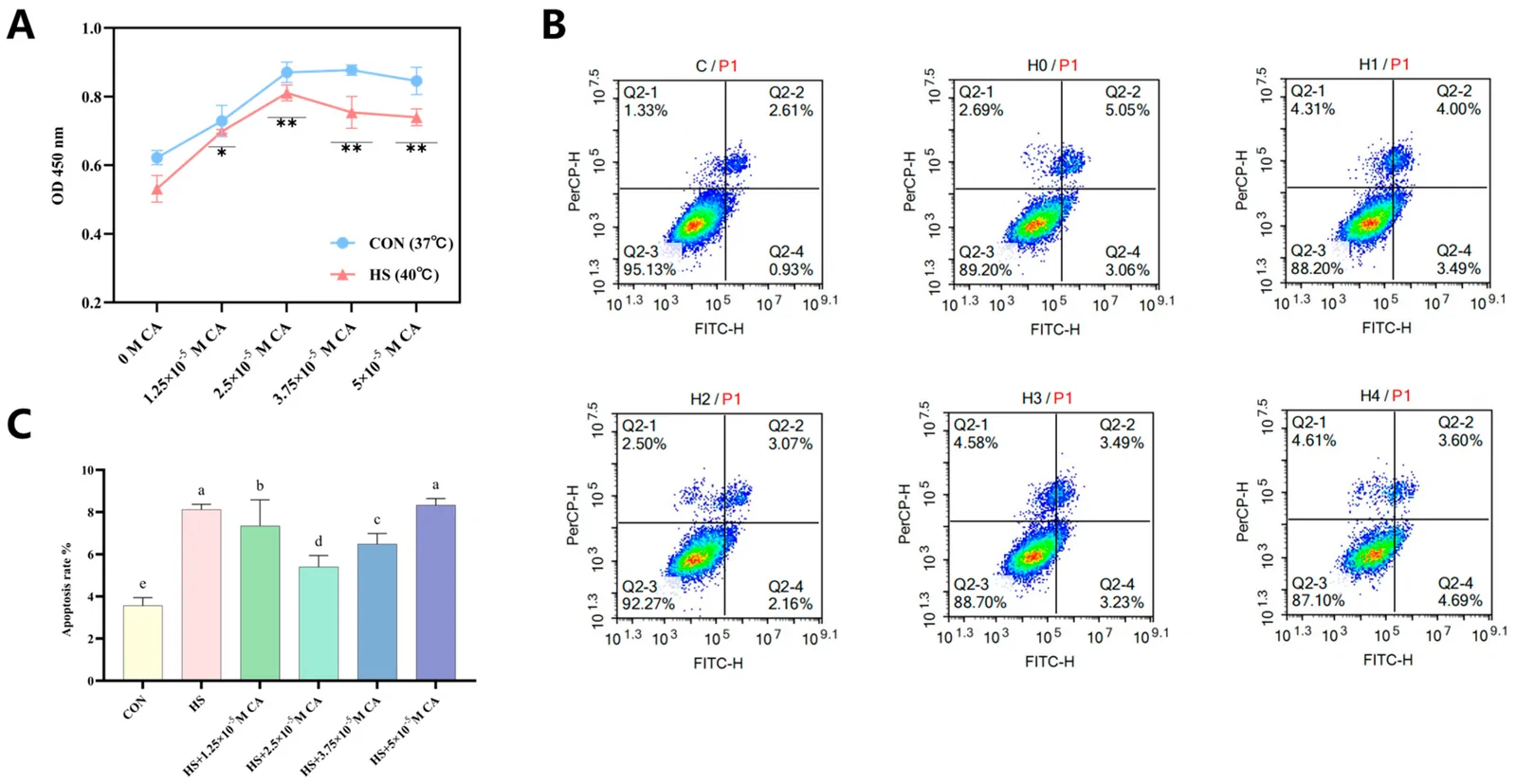
Effects of treatment with various CA concentrations on the viability of HS mouse follicular GCs: (**A**) CCK-8 assay. (**B**,**C**) Detection of cell apoptosis by flow cytometry.

Mouse follicular GCs were treated with different concentrations of CA at 37 °C and 40 °C according to experimental grouping. The results of cell viability detection are presented in Figure 6A. At both 37 °C and 40 °C, the cell viability was the highest in the group treated with CA at a concentration of 2.5 × 10^−5^ M compared with all other groups.

The cell apoptosis assay results are displayed in Figure 6B,C. The apoptosis rate of the HS group was extremely significantly higher than that of the CON group (*p* < 0.01). Compared with the HS group, the apoptosis rates of mouse follicular GCs in the 2.5 × 10^−5^ M and 3.75 × 10^−5^ M CA treatment groups decreased extremely significantly (*p* < 0.01). Considering the comprehensive effects of CA at various concentrations on the proliferation and apoptosis of GCs, CA at a concentration of 2.5 × 10^−5^ M was selected for cell treatment in subsequent experiments.

### 3.7. Effect of Cinnamaldehyde Treatment on E_2_ Secretion in Heat-Stressed Mouse Follicular Granulosa Cells

**Figure 7:**
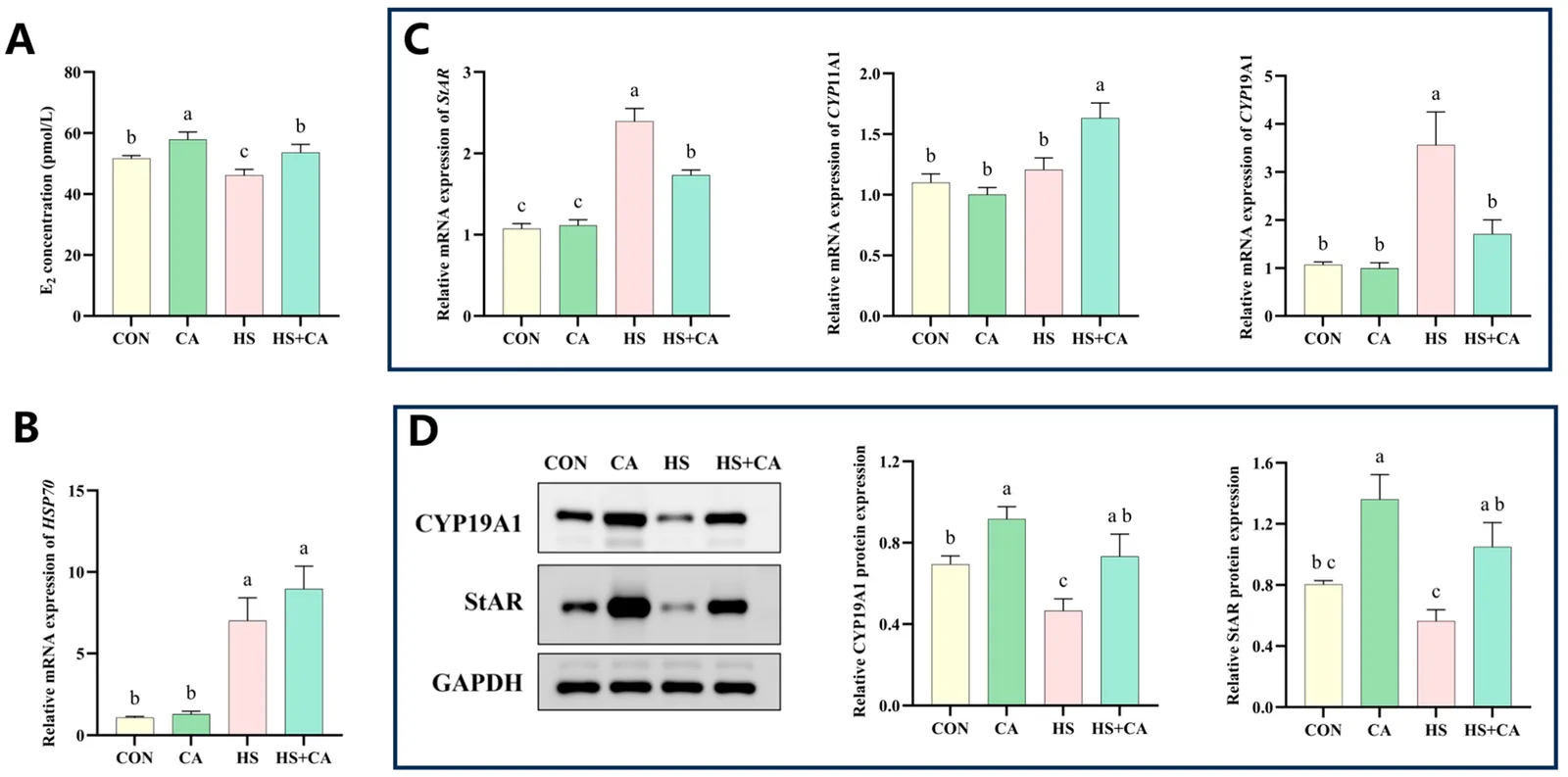
Effects of CA on E_2_ secretion and related proteins of follicular GCs in HS mice: (**A**) The secretion level of E_2_ was determined by ELISA. (**B**) Relative mRNA expression level of *HSP*70. (**C**) Effects of CA on the transcription of steroidogenic enzymes in follicular GCs of HS mice. (**D**) Effects of CA on the protein expression of steroidogenic enzymes in follicular GCs of HS mice.

CA elevated E_2_ secretion in mouse follicular GCs under HS (Figure 7A). Compared with the CON group, CA alone extremely significantly promoted E_2_ secretion of GCs (*p* < 0.01). E_2_ secretion of GCs was extremely significantly reduced under HS relative to the CON group (*p* < 0.01). E_2_ production was remarkably higher in the HS+CA group than in the HS group (*p* < 0.01). In conclusion, CA alleviates HS-induced impairment of E_2_ secretory function in GCs.

### 3.8. Effects of Cinnamaldehyde Treatment on HSP70 Gene in Heat-Stressed Mouse Follicular Granulosa Cells

The relative mRNA expression levels of *HSP*70 in each experimental group are shown in Figure 7B. The relative mRNA levels of *HSP*70 in the HS and HS+CA groups were extremely significantly higher than those in the CON and CA groups (*p* < 0.01). No significant difference was observed between the CA and CON groups.

### 3.9. Effects of Cinnamaldehyde Treatment on StAR and Steroidogenesis-Related Enzymes in Heat-Stressed Mouse Follicular Granulosa Cells

The transcriptional levels of steroidogenic acute regulatory protein (*StAR*) and steroidogenesis-related enzymes in each experimental group are presented in Figure 7C. Compared with the CON group, the relative mRNA expression levels of *StAR* and *CYP*19A1 were upregulated in mouse follicular GCs of the HS group. Relative to the HS group, the relative mRNA expression levels of *StAR* and *CYP*19A1 were significantly decreased in mouse follicular GCs of the HS+CA group (*p* < 0.05); meanwhile, the relative mRNA level of *CYP*11A1 was significantly increased in the HS+CA group versus the HS group (*p* < 0.05).

The protein expression levels of StAR and CYP19A1 in all experimental groups are shown in Figure 7D. Compared with the CON group, treatment with CA alone significantly elevated the protein levels of CYP19A1 and StAR. In the HS group, the protein expression of CYP19A1 was significantly reduced relative to the CON group (*p* < 0.05), while StAR protein expression exhibited a declining trend without statistical significance. Compared with the HS group, the protein abundances of StAR and CYP19A1 were extremely significantly upregulated in the HS+CA group (*p* < 0.01).

## 4. Discussion

HS is one of the prevalent environmental stressors in livestock and poultry farming in high-temperature and high-humidity regions [22]. HS severely impairs growth performance, reproductive capacity, and egg-laying performance of livestock and poultry [23,24,25]. Alleviating HS injuries in mammals with plant extracts has become a popular research focus in recent years [26,27].

In this study, CA was used to treat HS mice and mouse follicular GCs subjected to HS, aiming to clarify the effects of CA on ovarian function in HS mice. Subsequent experiments were carried out after determining the administration dosage. During CA treatment of HS mice, body weight and E_2_ secretion levels were monitored at three time points: W1, W2, and W3. The results indicated that there were no significant differences in mouse body weight among all experimental groups at W1 and W2, whereas body weight showed a downward trend in the HS group and HS+DMSO group. By W3, body weights of mice in the HS group and HS+DMSO group were significantly decreased. These findings indicated that prolonged chronic HS restricts body weight gain in animals. This phenomenon may be attributed to HS-induced disturbances in appetite as well as intestinal digestion and absorption capacity [28]. Meanwhile, we observed that compared with the HS group and HS+DMSO group, mice in the HS+CA group exhibited an upward trend in body weight, with body weight recovering to a level with no statistically significant difference relative to the CON group. This may be because CA alleviates HS-triggered oxidative stress and intestinal injury [29,30]. This could effectively reduce unnecessary energy consumption in the body and improve intestinal absorption function. E_2_ detection results at W1, W2, and W3 indicated that CA could effectively elevate E_2_ secretion levels in HS mice. On the other hand, ovarian histological results revealed that cumulative chronic HS altered ovarian histological morphology over time. Massive follicular atresia occurred, follicular development was arrested at the primordial and primary stages, and the proportion of mature functional follicles decreased, ultimately leading to ovarian dysfunction. CA intervention significantly increased the proportion of secondary and antral follicles and ameliorated ovarian function. Collectively, CA effectively alleviated HS-induced reproductive damage in female mice in animal experiments.

Mouse follicular GCs are vital somatic cells surrounding oocytes. They exert crucial functions throughout the entire follicular developmental cycle and serve as one of the essential cell types sustaining normal ovarian physiological functions. Using mouse follicular GCs as the research object, we further explored the mechanisms by which CA alleviates HS damage in mice. GCs were treated with different concentrations of CA. Cell viability and apoptosis rate were detected using the CCK-8 assay and flow cytometry, respectively. Ultimately, CA at 2.5 × 10^−5^ M was confirmed as the optimal treatment concentration for subsequent cell experiments. During the screening of CA concentrations, we found that CA could effectively alleviate HS-induced cellular apoptosis. This may be one of the critical factors accounting for the elevated E_2_ secretion in HS mice treated with CA. E_2_ concentrations were further quantified at the cellular level. The results demonstrated that CA increased E_2_ secretion in mouse follicular GCs under HS. This outcome was consistent with the findings from animal experiments, confirming that CA effectively ameliorates the HS-induced reduction in E_2_ secretion. This conclusion implies that CA holds promising potential for protecting the reproductive system of female mammals and sustaining ovarian development.

HSP70 is a canonical biomarker of HS in organisms [31]. HS triggers compensatory upregulation of HSP70 at both transcriptional and translational levels in cells. HSP70 mediates the degradation of misfolded proteins and inhibits cellular apoptosis, acting as an endogenous stress-responsive molecule that enables cells to counteract high-temperature damage [32]. Notably, we found that the separate addition of CA at 37 °C caused no change in the relative mRNA expression level of *HSP*70. Meanwhile, CA treatment failed to downregulate *HSP*70 mRNA expression under HS. These results suggest that the protective effect of CA on GCs is unlikely to be achieved via inhibiting *HSP*70 transcription.

E_2_ biosynthesis is highly dependent on steroidogenic enzymes. StAR, CYP11A1, and CYP19A1 jointly promote E_2_ secretion through three successive steps: substrate transportation, precursor generation, and terminal conversion [33,34,35]. In the present study, we observed an interesting phenomenon: supplementation with CA alone at 37 °C exerted no significant effects on the relative mRNA expression levels of *StAR*, *CYP*11A1, and *CYP*19A1, yet markedly elevated the relative protein expression levels of StAR and CYP19A1. This may be related to the increased translational efficiency of ribosomes. More importantly, HS significantly upregulated the relative mRNA expression levels of *StAR* and *CYP*19A1, whereas the relative protein expression levels of StAR and CYP19A1 were markedly decreased. This phenomenon may represent a mechanism by which HS disrupts cellular homeostasis. Impaired endoplasmic reticulum function and activation of the unfolded protein response (UPR) trigger global translational arrest in cells [9]. Therefore, we speculate that this phenomenon may arise because the lack of downstream E_2_ triggers a feedback-mediated increase in the transcription of synthase genes; these transcripts cannot be efficiently translated into functional proteins, ultimately hindering E_2_ synthesis under HS. Notably, treatment with CA at 2.5 × 10^−5^ M successfully alleviated this phenomenon. After CA administration, the protein expression levels of StAR and CYP19A1 were significantly elevated in HS mouse ovarian follicular GCs, eventually restoring E_2_ secretion of mouse follicular GCs to normal physiological levels.

As a natural plant extract, this study confirmed that CA sustains E_2_ secretion by upregulating the expression of steroidogenic enzymes and effectively alleviates HS-induced reproductive damage in female mice. This finding provides a theoretical basis for the further application of CA to counteract HS in female mammals.

## 5. Conclusions

Animal experiments showed that intragastric administration of CA at 25 mg/kg caused no obvious hepatotoxicity. This dosage effectively ameliorated HS-induced ovarian histological injury and elevated serum E_2_ concentrations under both normal and HS conditions. Further in vitro cellular assays verified that treatment with 2.5 × 10^−5^ M CA markedly increased cell viability and suppressed excessive apoptosis in mouse follicular GCs exposed to HS. Under HS, CA boosted E_2_ secretion in mouse follicular GCs by upregulating the protein expression of StAR and CYP19A1.

## Figures and Tables

**Table 1:** Histological characteristics of follicles at various stages.

Follicle Type	Follicular Characteristics
Primordial follicle	It is composed of an oocyte surrounded by a single layer of flattened precursor granulosa cells
Primary follicle	It is composed of an oocyte surrounded by a single layer of cuboidal granulosa cells
Secondary follicle	It is composed of an oocyte surrounded by two or more layers of cuboidal granulosa cells
Antral follicle	It is composed of an oocyte surrounded by multiple layers of cuboidal granulosa cells, with visible formation of the follicular antrum and cumulus oophorus

**Table 2:** Primer sequences.

Primer Name	Primer Sequence (5′→3 ′ )	Amplicon Size (bp)	GenBank Number
*β-actin*	F: GCAAGCAGGAGTACGATGAG R: CCATGCCAATGTTGTCTCTT	148	NM_007393.5
*HSP*70	F: TTCGAGGGCATCGACTTCTACAC R: AACAGGTCCGAGCACAGCTCTT	69	NM_010478.3
*StAR*	F: CTTGGCTGCTCAGTATTGAC R: TGGTGGACAGTCCTTAACAC	153	NM_011485.5
*CYP*19A1	F: GACACATCATGCTGGACACC R: CAAGTCCTTGACGGATCGTT	179	NM_007810.4
*CYP*11A1	F: CGATACTCTTCTCATGCGAG R: CTTTCTTCCAGGCATCTGAAC	126	NM_019779.4

## Data Availability

The data presented in this study are available upon request from the corresponding author. The data are not publicly available due to privacy or ethical restrictions.

## Supplementary Materials

The following supporting information can be downloaded at https://www.mdpi.com/article/10.3390/ani16213512/s1, Figure S1: Full-length blot; Figure S2: Vaginal smear of mice at estrus. Abundant anucleated cornified epithelial cells are visible, and no leukocytes are observed.

## Abbreviations

The following abbreviations are used in this manuscript:

StAR	Steroidogenic acute regulatory
CYP11A1	Cytochrome P450 family 11 subfamily A member 1
CYP19A1	Cytochrome P450 family 19 subfamily A member 1
RT-qPCR	Real-time quantitative reverse transcription PCR
SDS-PAGE	Sodium dodecyl sulfate-polyacrylamide gel electrophoresis
PBS	Phosphate-buffered saline
BSA	Bovine serum albumin
FSHR	Follicle-stimulating hormone receptor
DAPI	4′,6-diamidino-2-phenylindole
DMSO	Dimethyl sulfoxide
ELISA	Enzyme-linked immunosorbent assay
