# Supplementary material for: Protective Effects of Cinnamaldehyde Against Heat Stress in Mice and Its Regulatory Influence on Estrogen Secretion
Source: Animals (Basel). 2026 Nov 6;16(21):3512. doi: 10.3390/ani16213512 (PMC13761615; doi:10.3390/ani16213512)
Supplement: Supplementary file 1 [file animals-16-03512-s001.zip › animals-4583960-supplementary.pdf]

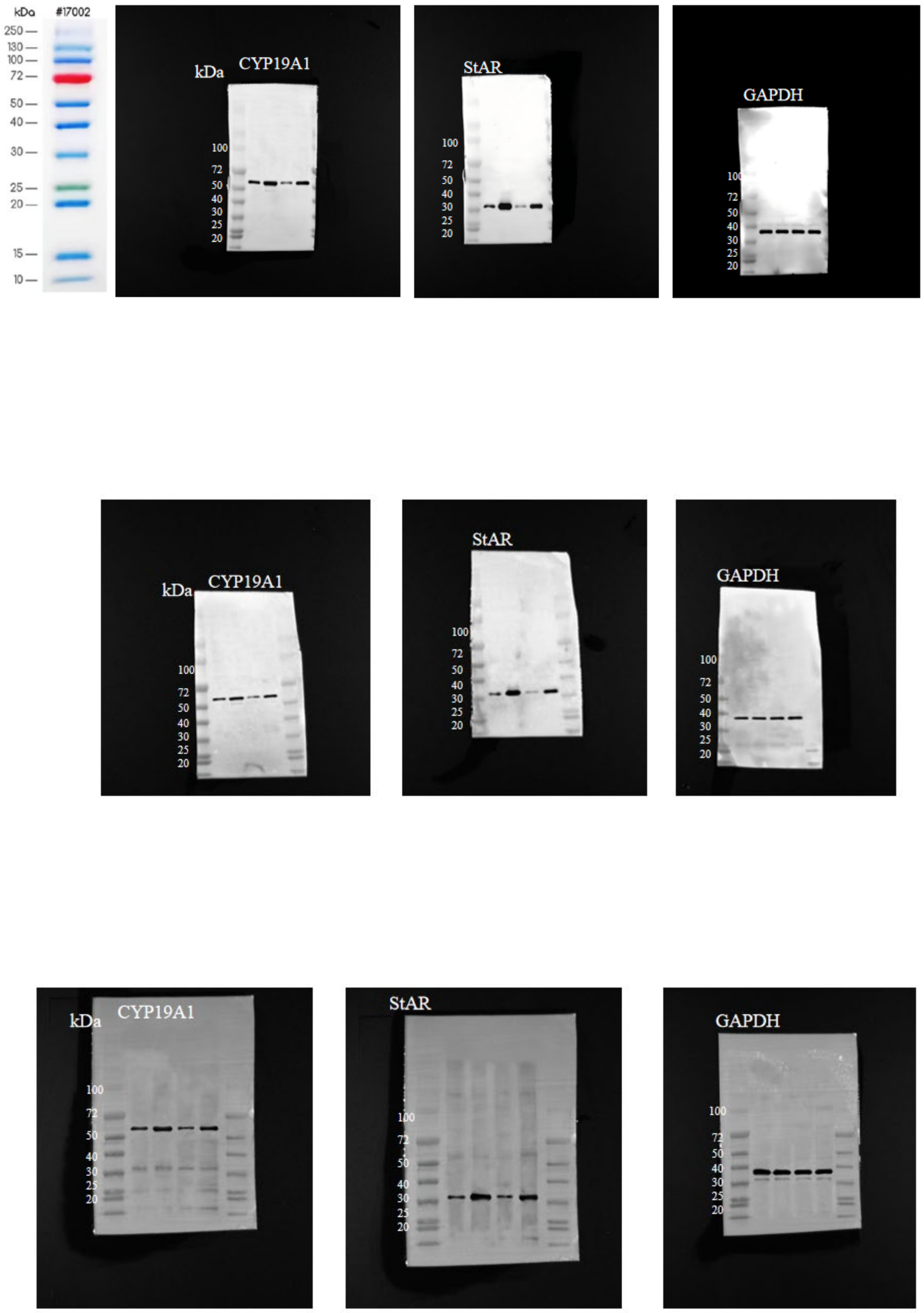

Figure S1. Full-length blot

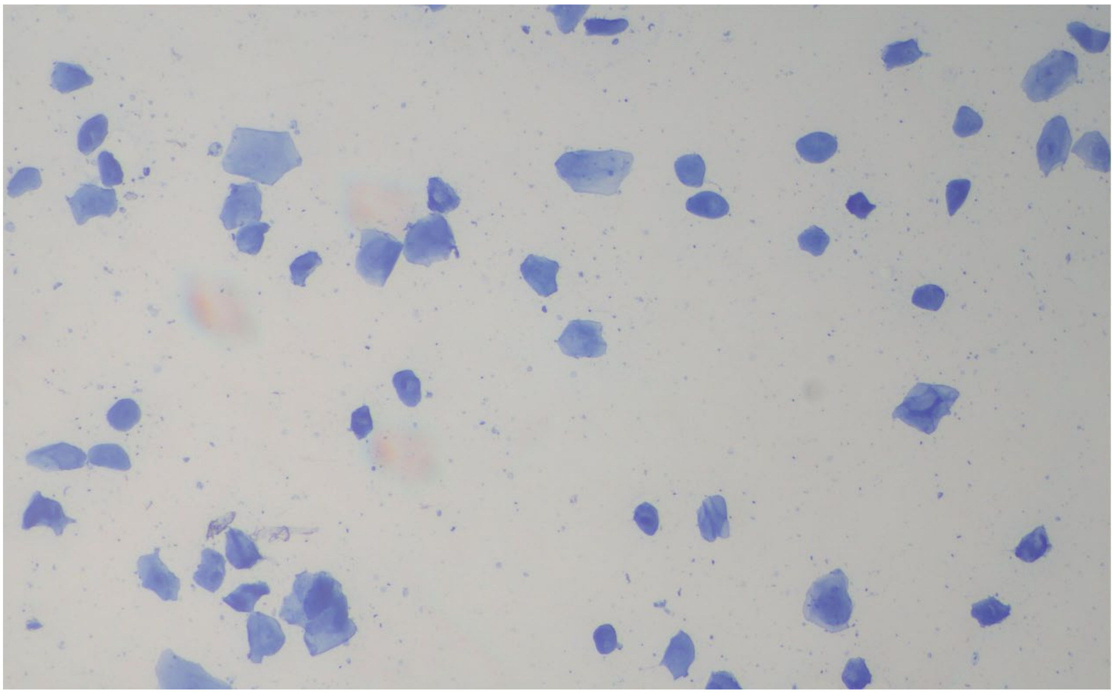

Figure S2. Vaginal smear of mice at estrus. Abundant anucleated cornified epithelial cells are visible, and no leukocytes are observed.
